# Chronic Infection with Hidden Malignancy Mimicking the Clinical Presentation of an Autoimmune Disease

**DOI:** 10.1155/2022/5392858

**Published:** 2022-07-18

**Authors:** Ammar Bakhsh, Khalil Miyajan, Abdulghani Sadaqa, Amer Eisa, Ghali Faidah, Hani Almoallim

**Affiliations:** ^1^Department of Medicine, Faculty of Medicine, Umm Alqura University, Makkah, Saudi Arabia; ^2^Department of Medicine, Dr. Sameer Abbas Hospital, Jeddah, Saudi Arabia

## Abstract

Infections of the paranasal sinuses are common and usually occur in patients who are immunocompromised. Many atypical clinical presentations have been reported but rarely in the elderly population. We report a 71-year-old female patient with a 20-year history of an autoimmune disease who had recently become resistant to treatment. Her autoimmune symptoms significantly improved following resection of deeply seated bacterial infection in her paranasal sinuses. She was also diagnosed with cervical cancer. Clinicians should look carefully for hidden infections and/or malignancies in patients lacking response while on immunosuppressive therapy for autoimmune disease.

## 1. Introduction

Autoimmune diseases (ADs) are categorized by intrinsic immune modifications that may lead to chronic inflammation in multiple organ systems [1]. Many pathogenic mechanisms contribute to the development of these diseases. Chronic infection has been described as a common precipitant of autoimmune phenomena [2]. Additionally, malignancies have been linked to several ADs [3]. The diseases themselves and many of their treatments are linked to an increased risk for severe infection [4].

Infectious pathogens (bacteria, viruses, and parasites) can initiate or exacerbate a strong immune response and possibly disrupt balance and regulation, leading to autoimmunity. The strongest evidence to support this theory would be the diminishing of symptoms following clearance of an infection.

We report a case of a chronic persistent autoimmune clinical manifestation in an elderly female patient. Her symptoms significantly improved after surgical clearance of deeply seated bacterial infections in her paranasal sinuses. She was later diagnosed with localized cervical cancer which was resected.

## 2. Case Report

A 71-year-old female patient, with uncontrolled asthma, was a known case of rheumatoid arthritis (RA) since 2004. She presented to the outpatient rheumatology clinic in February 2019 complaining of easy fatigability with body aches for six months. This was associated with episodic unilateral recurrent eye and facial swelling, sinusitis, cough, shortness of breath, and skin rash. Her physical examination upon presentation showed stable vital signs. She had left knee tenderness, with a skin rash on an erythematous base over the left knee. The remainder of her physical examination was unremarkable. Her initial bloodwork at that time showed the following: high inﬂammatory marker—C-reactive protein (CRP) of 35.4 (0–3.0 (mg/L)), and negative antinuclear antibody (ANA) initially in an outside laboratory. Additional investigations in subsequent visits, however, revealed ANA of 197.3 (<20 unit), Anti-SSB 97.5 (<20 unit), Anti-SSA 69.6 (<20 unit), and negative serology for anti-double strand DNA, rheumatoid factor, and anti-citrullinated peptide antibody. Her chest and upper neck computed tomography (CT) scan revealed bilateral upper lobe peripheral branching densities consistent with small airway disease with mucous impaction, as well as a 4 mm calcified granuloma on the right lower lobe and bilateral posterior basal cystic bronchiectasis with multiple scattered pneumatoceles and minimal apical parenchyma without any concerning pulmonary nodules. The provisional diagnosis was autoimmune phenomena probably related to systematic lupus erythematosus (SLE). Thus, she was started on 200 mg hydroxychloroquine twice daily.

In April 2019, she presented again with episodic attacks, this time with non-itchy erythematous patches in her legs and arms. Her episodic unilateral eye and facial swelling with redness persisted as well during these attacks. Her bloodwork showed high CRP 63.5 (0–3.0 (mg/L)) with increasing white blood cells 11.18 (4.5–11 (10^3/uL)), and an arranged biopsy of her skin patches revealed subepidermal blistering disease with neutrophils. These findings were consistent with a few diagnoses: sweet syndrome, dermatitis, and a pyodermal disorder. Consequently, she was started on mycophenolate mofetil 1000 milligram (mg), colchicine 0.50 mg, and continued hydroxychloroquine 200 mg twice daily. Her symptoms, however, only improved with short courses of systemic steroids.

In August 2019, she presented again with a new episode of recurrent unilateral facial swelling which subsided prior to her clinical visit. Additionally, she was diagnosed with primary bilateral conductive hearing loss, but her otorhinolaryngology consultation did not raise any further concerns. Physical examination showed fine erythematous spots on her scalp. Her CRP was 18.61 (0–3.0 (mg/L)). Consequently, an X-ray skeletal survey showed multiple lytic lesions in the skull. Urine and serum protein electrophoresis was negative, and CT staging was normal. An oncology referral was considered, and further workup was initiated. As a result, she was initially treated with prednisone 30 mg once daily for six days. Over the following months, her symptoms varied in severity ranging from almost no symptoms to aggressive eye and facial swelling with redness. Mycophenolate was switched to 10 mg of weekly methotrexate. Her episodic attacks were mainly controlled with short courses of prednisone. Her hydroxychloroquine 200 mg twice daily was maintained.

**Figure 1:**
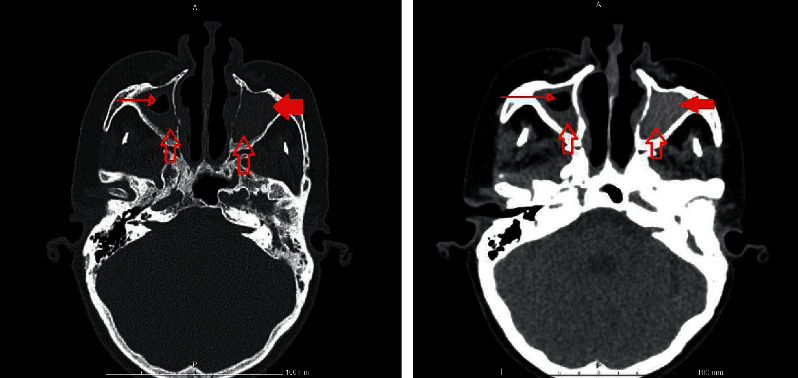
Axial paranasal CT showed significant hyper-density in both maxillary sinuses (arrows) with completely obliterated left maxillary sinus (dense arrow) and air-fluid level within right maxillary sinus (small arrow) likely related to an underlying infection.

In July 2020, sinus CT scans were performed due to frontal headache associated with nasal discharge. This was in addition to her usual episodic attacks of a unilateral eye and facial swelling with redness. The CT scan revealed right frontal dehiscence with bilateral maxillary sinus opacification (Figure 1). The final diagnosis was chronic sinusitis. She underwent fundal endoscopic sinus surgery (FESS), and a deeply seated *Citrobacter freundii* overgrowth was resected. She was maintained on ciprofloxacin 500 mg twice daily for two weeks and followed closely by her surgeon until she completed a full course. Following this, the patient had complete resolution of her symptoms and no longer required any of the medications she was taking previously except for a tapering regimen of prednisone.

**Figure 2:**
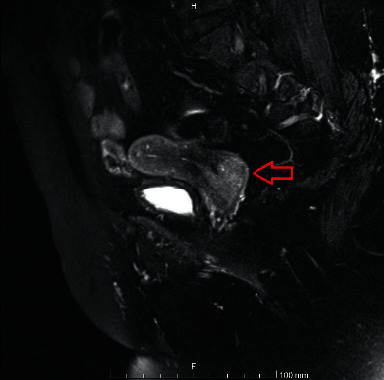
Pelvic MRI showed a lower cervical mass with transmural stromal involvement measuring 4 × 4 × 3 cm (arrow). It is protruding to the upper half of the vagina and expanding vaginal fornices.

In October 2020, she presented again with vaginal spotting and a 3-4 centimeter (cm) cervical mass per vaginal examination. The oncology workup revealed a positive pap smear for atypical cells. The biopsy showed non-keratinizing poorly differentiated invasive squamous-cell carcinoma, and a pelvic MRI revealed a large lower cervical mass protruding to the upper half of the vagina measuring up to 4 cm in the two largest dimensions without parametrial invasion that staged llA2 by FIGO [5] (Figure 2). Her steroid dose was tapered to a complete stop. She came to follow-up two months after her surgery without any of her usual autoimmune clinical presentations. The oncology service took over the care of this patient.

## 3. Discussion

We report a case of episodic and recurrent autoimmune clinical presentations in an elderly female patient. This was in the form of unilateral eye and facial swelling with redness, neutrophilic skin eruption, and, lastly, frontal headaches and nasal discharge. There was a background diagnosis of RA since 2004. There was a lack of response to several immunosuppressive therapies. Prednisone was the only drug that could control these episodic attacks. These attacks disappeared following the resection of deeply seated bacterial infection in her paranasal sinuses. Following extensive workup to rule out malignancy, this patient was diagnosed with cervical cancer.

This case suggests that ADs not responding to treatment in the elderly age group can be associated with hidden infections and/or malignancy. This is in accordance with other reports of patients with disseminated sporotrichosis who were misdiagnosed as having RA, gout, or psoriatic arthritis. These patients had multiple treatment failures with systemic immunosuppressive drugs [6–9]. This demonstrates how chronic untreated bacterial infections may mimic autoimmune inflammatory disorders. Also, the diagnosis of hidden bacterial infection can be a real challenge requiring thorough investigations, as seen in our patient who had *Citrobacter freundii* which is unusual in chronic maxillary sinusitis [10].

The patient in our report was ultimately diagnosed with cancer of the cervix, and she is currently following with an oncology service. She had elevated inflammatory markers, which could have been due to an AD, infection, malignancy, or combined factors. The relationship between these manifestations is a venue for further research. The association between malignancies and ADs has abundant findings in the literature yet remains poorly understood. Chronic inflammation and cellular damage from autoimmune responses are theorized to be strongly associated with cancer development. Essentially, the continuous elevation of inflammatory markers may be associated with an increased risk for malignancies [11]. Our case highlights the importance of detailed workup, especially for patients with persistent autoimmune symptoms and a lack of response to treatment.

The relationship between ADs, chronic infection, and cancer is complicated and can be tri-directional. In patients with different ADs, the risk of infection and hematological and non-hematological malignancies increases [12, 13]. On the other hand, some cancers may increase the risk of developing an autoimmune disorder [13, 14]. Also, long-term exposure to pathogens or opportunistic organisms in the environment causes an abnormal immune response to self-antigens, resulting in systemic or organ-specific damage that may initiate or exacerbate ADs [2, 13, 15]. In our case, we were unable to identify the exact lines between these issues. What we had observed was the total resolution of all AD manifestations following the resection of a bacterial infection. Was this patient suffering from true AD, or was it the result of an interaction between infection and/or malignancy that had been present since 2004?

Cancers may present with clinical features similar to an AD, such as RA, SLE, Sjogren's syndrome (SS), scleroderma (Sc), and other ADs [16]. SLE and RA have been linked to persistent human papillomavirus (HPV) infection and increased risk of high-grade cervical dysplasia and cervical cancer [17]. This may be due to an immunocompromised state from the AD, the immunosuppressive medications used like glucocorticoid, or effects from both that can lead to disseminated HPV infection [18, 19]. This could explain why our patient developed cervical cancer. The mechanism by which HPV infection can progress to cervical cancer among immunosuppressed patients can be explained by impaired immune responses. This may lead to an inability or decrease in the clearance of HPV infection, resulting in persistent infection and increased risk of high-grade infection dysplasia and cervical cancer [17].

We want to raise physician's awareness of the presence of hidden infection and/or malignancy, especially in patients with high-risk factors such as old age, immunosuppressive medication, and chronic inflammation. This should be clearly emphasized in elderly age group patients with autoimmune manifestation with a lack of proper response to treatment.

## Data Availability

All data used to support the findings of this study are available upon request to the corresponding author.

## References

[1] Ercolini A. M., Miller S. D. (2008). The role of infections in autoimmune disease. *Clinical and Experimental Immunology*.

[2] Kivity S., Agmon-Levin N., Blank M., Shoenfeld Y. (2009). Infections and autoimmunity–friends or foes?. *Trends in Immunology*.

[3] Abu-Shakra M., Buskila D., Ehrenfeld M., Conrad K., Shoenfeld Y. (2001). Cancer and autoimmunity: autoimmune and rheumatic features in patients with malignancies. *Annals of the Rheumatic Diseases*.

[4] Singh J. A., Hossain A., Kotb A., Wells G. (2016). Risk of serious infections with immunosuppressive drugs and glucocorticoids for lupus nephritis: a systematic review and network meta-analysis. *BMC Medicine*.

[5] Tsikouras P., Zervoudis S., Manav B. (2016). Cervical cancer: screening, diagnosis and staging. *Journal of Buon*.

[6] Yamaguchi T., Ito S., Takano Y. (2012). A case of disseminated sporotrichosis treated with prednisolone, immunosuppressants, and tocilizumab under the diagnosis of rheumatoid arthritis. *Internal Medicine*.

[7] Gottlieb G. S., Lesser C. F., Holmes K. K., Wald A. (2003). Disseminated sporotrichosis associated with treatment with immunosuppressants and tumor necrosis factor-*α* antagonists. *Clinical Infectious Diseases*.

[8] Chowdhary G., Weinstein A., Klein R., Mascarenhas B. R. (1991). Sporotrichal arthritis. *Annals of the Rheumatic Diseases*.

[9] Appenzeller S., Amaral T. N., Amstalden E. M. I. (2006). Sporothrix schenckii infection presented as monoarthritis: report of two cases and review of the literature. *Clinical Rheumatology*.

[10] Olszewski J., Miłoński J. (2008). The analysis of the bacterial and fungal flora in maxillary sinuses in patients operated due to FESS method. *Otolaryngologia Polska*.

[11] Cappelli L. C., Shah A. A. (2020). The relationships between cancer and autoimmune rheumatic diseases. *Best Practice and Research Clinical Rheumatology*.

[12] Bernatsky S., Ramsey-Goldman R., Clarke A. E. (2009). Malignancy in systemic lupus erythematosus: what have we learned?. *Best Practice and Research Clinical Rheumatology*.

[13] Arango M.-T., Shoenfeld Y., Cervera R., Anaya J.-M. (2013). *Autoimmunity: From Bench to Bedside*.

[14] Tsuzuki S., Takahashi H., Chen F., Setoguchi K. (2019). *Features of Autoimmune Disorder of Cancer Patients*.

[15] Chakravarty E. F. (2008). Viral infection and reactivation in autoimmune disease. *Arthritis and Rheumatism*.

[16] Giat E., Ehrenfeld M., Shoenfeld Y. (2017). Cancer and autoimmune diseases. *Autoimmunity Reviews*.

[17] Kim S. C., Glynn R. J., Giovannucci E. (2015). Risk of high-grade cervical dysplasia and cervical cancer in women with systemic inflammatory diseases: a population-based cohort study. *Annals of the Rheumatic Diseases*.

[18] Ko M.-J., Chu C.-Y. (2004). Disseminated human papillomavirus type 11 infection in a patient with pemphigus vulgaris: confirmed by DNA analysis. *Journal of the American Academy of Dermatology*.

[19] Feldman C. H., Kim S. C. (2014). Should we target patients with autoimmune diseases for human papillomavirus vaccine uptake?. *Expert Review of Vaccines*.

